# A kinematic study on (un)intentional imitation in bottlenose dolphins

**DOI:** 10.3389/fnhum.2015.00446

**Published:** 2015-08-05

**Authors:** Luisa Sartori, Maria Bulgheroni, Raffaella Tizzi, Umberto Castiello

**Affiliations:** ^1^Dipartimento di Psicologia Generale, Università di PadovaPadova, Italy; ^2^Cognitive Neuroscience Center, Università di PadovaPadova, Italy; ^3^Ab.Acus. S.r.l.Milano, Italy; ^4^Oceanomare Delphis OnlusRimini, Italy; ^5^Centro Interdisciplinare Beniamino Segre, Accademia dei LinceiRoma, Italy

**Keywords:** imitation, mimicry, bottlenose dolphins, mirror neurons, automatic imitation, visuomotor priming

## Abstract

The aim of the present study was to investigate the effect of observing other’s movements on subsequent performance in bottlenose dolphins. The imitative ability of non-human animals has intrigued a number of researchers. So far, however, studies in dolphins have been confined to intentional imitation concerned with the explicit request to imitate other agents. In the absence of instruction to imitate, do dolphins (un)intentionally replicate other’s movement features? To test this, dolphins were filmed while reaching and touching a stimulus before and after observing another dolphin (i.e., model) performing the same action. All videos were reviewed and segmented in order to extract the relevant movements. A marker was inserted *post hoc* via software on the videos upon the anatomical landmark of interest (i.e., rostrum) and was tracked throughout the time course of the movement sequence. The movement was analyzed using an in-house software developed to perform two-dimensional (2D) *post hoc* kinematic analysis. The results indicate that dolphins’ kinematics is sensitive to other’s movement features. Movements performed for the “visuomotor priming” condition were characterized by a kinematic pattern similar to that performed by the observed dolphin (i.e., model). Addressing the issue of spontaneous imitation in bottlenose dolphins might allow ascertaining whether the potential or impulse to produce an imitative action is generated, not just when they intend to imitate, but whenever they watch another conspecific’s behavior. In closing, this will clarify whether motor representational capacity is a by-product of factors specific to humans or whether more general characteristics such as processes of associative learning prompted by high level of encephalization could help to explain the evolution of this ability.

## Introduction

The human capacity for imitation is well-known and is considered one of the hallmarks of human cognition and culture (Meltzoff and Prinz, 2002). From infancy on, humans habitually copy behaviors of every type across a variety of contexts (Meltzoff, 1996; Tomasello, 1999). For many decades, those studying imitation in children and animals focused on *intentional* imitation because it was thought that imitation must be controlled in order to play an important role in cognitive and social development, and to mediate cultural inheritance (Heyes, 2010).

Given the central role of imitation in human cognitive evolution and development, it is perhaps not surprising that a great amount of research has been devoted to ascertain whether this ability is shared among non-human animals (Galef, 1988; Fiorito and Scotto, 1992; Whiten and Ham, 1992; Leggio et al., 2000; Byrne and Bates, 2010).

A surprising aspect stemming from this body of work is that the animal most similar to humans in imitative abilities may not be found among our closest relatives, but rather among cetaceans (Herman, 2002). For instance, bottlenose dolphins (*Tursiops truncatus*) exhibit a prominent ability to copy motor behaviors of conspecifics, humans, and other animals (Tayler and Saayman, 1973; Bauer and Johnson, 1994; Herman, 2002; Jaakkola et al., 2010). So far, imitation in dolphins has been chiefly investigated in terms of explicit request to imitate (e.g., Herman, 1980; Herman et al., 1989; Marino, 2002; Kuczaj and Highfill, 2005; Kuczaj and Walker, 2006). Rather, mimicry, imitative behavior that is *not intended* (Stürmer et al., 2000) and of which the imitator may be unaware (Chartrand and Bargh, 1999), has received little attention. Unintentional imitation is a compatibility effect in which the speed and/or accuracy of behavioral performance is modulated by the relationship between the topographic features of an observed action and the observer’s responses. In the absence of instruction to imitate, movement observation *facilitates* execution of the observed action. Such facilitation effects have been described in humans as a decrease in reaction time and an increase in average velocity when an observed and a subsequently executed hand action matched (i.e., *Visuomotor Priming*; see Craighero et al., 1998; Castiello et al., 2002; Edwards et al., 2003; Heyes et al., 2005). It is generally agreed that unintentional imitation effects result from a process in which action observation activates motor representations that are “similar” to the action observed (Heyes, 2011). The interesting question is then: does unintentional imitation depends on learned or genetically prespecified, stimulus-response (S-R) connections? Single-unit recording indicates that monkeys have mirror neurons in the premotor cortex, that is, cells that fire during observation and execution of the same action (di Pellegrino et al., 1992; Gallese et al., 1996). Similarly, the areas of the human brain that are activated by observation and execution of the same actions are sometimes called the “mirror neuron system” (Gazzola and Keysers, 2009; Kilner et al., 2009). Many developmental and comparative psychologists have suggested that nonhuman primates show “mimicry” (Tomasello, 1996; Meltzoff and Moore, 1997) or “response facilitation” (Byrne and Russon, 1998) because of simple, innate S-R links, and it is widely assumed that the perception–action matching properties of “mirror neurons” are present at birth (Ferrari et al., 2009). On the other hand, two theoretical accounts explain unintentional imitation as a by-product of learned associations between perceptual and motor representations: the ideomotor theory of action control (Prinz, 2005; Massen and Prinz, 2009) and the associative sequence learning model of imitation (Heyes et al., 2005; Catmur et al., 2009). In this connection, the extent to which animals can imitate might reflect representational capacity and is a matter of considerable importance (Whiten, 1998; Suddendorf and Whiten, 2001; Kuczaj and Yeater, 2006). This is one reason why studying unintentional imitation in dolphins is particularly significant. Conclusive evidence of unintentional imitation in a species as phylo-genetically distant from primates as dolphins would play a pivotal role in determining whether this motor representational capacity is a by-product of factors specific to primates or whether more general characteristics such as processes of associative learning related to a high level of encephalization could help to explain the evolution of this ability.

To investigate this issue, here we adapted a classic visuomotor priming paradigm. A dolphin “A” observed another dolphin (i.e., model) performing a reach-to-touch (with the rostrum) action towards a spherical object. Subsequently, the dolphin “A” performed the same action towards the same object. A control condition in which the dolphin performed the same movement in the absence of any motor priming was also included. On the basis of previous human findings based on a similar paradigm (e.g., Edwards et al., 2003; Pierno et al., 2008), we hypothesize that if the trailing dolphin is facilitated when primed by a dolphin model, then reach-to-touch movement should exhibit a higher average velocity than when visuomotor priming does not occur. Conversely, if no facilitation occurs, then primed and non-primed movements should not differ from a kinematical perspective.

## Materials and Methods

### Participants

**Figure 1:**
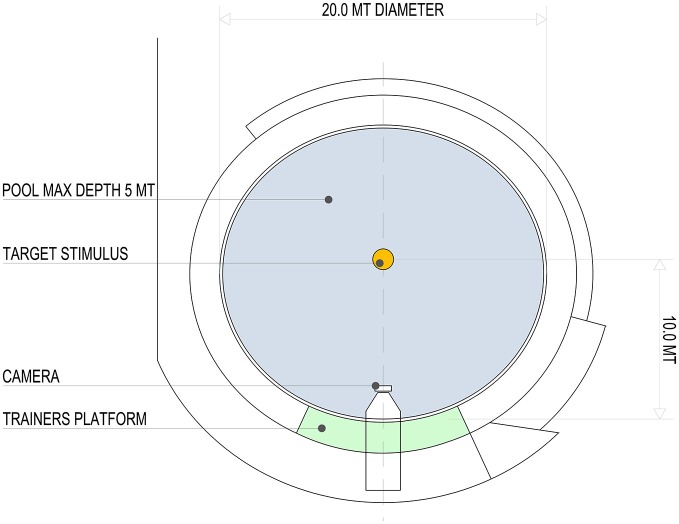
**Aerial view of the pool (20 m in diameter; capacity 1300 m^3^, surface 310 m^2^ and maximum depth of 5 m) and relative position of the target stimulus, video camera and trainer’s platform**.

Two adult bottlenose dolphins (*Tursiops truncatus*, S. and L., male and female, 20 and 21 years respectively) participated in the study. They swam in a pool of a round shape (20 m in diameter; capacity 1300 m^3^, surface 310 m^2^ and maximum depth of 5 m; Figure 1). The experimental procedure for the dolphins was approved by the committee for animal research of the University of Padova and adhered to the *Ethical Guidelines for the Conduct of Research on Animals by Zoos and Aquariums* issued by the World Association of Zoos and Aquariums (WAZA).

### Stimulus and Experimental Set Up

**Figure 2:**
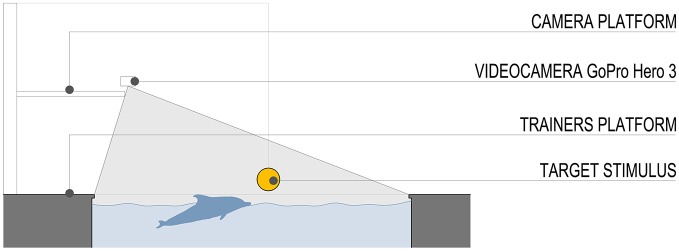
**Schematic section of the pool and field of view of the video camera**. The stimulus was a plastic ball (diameter 20 cm) attached to a pinnacle located to a fixed distance of 10 m from the starting position.

The stimulus was a plastic ball (diameter 20 cm) attached to a pinnacle located to a fixed distance of 10 m from the starting position (Figure 2). Both the dolphins participating in this experiment had already familiarized and played with the plastic ball attached to the pinnacle in previous years. We purposely abstained from introducing a new stimulus in the pool area to avoid possible learning effects.

### Video Recording Technique

A total of 12 h of video footage was filmed during daylight hours between 10.00 a.m. and 12.00 p.m. in the time period between 27th of May and 7th of June, 2013. The video was filmed *ad libitum* using a A GoPro camera Hero3 (Black Edition Wi-Fi compatible) fastened to a rigid trampoline above the plane of motion. This procedure was utilized to guarantee a constant point of reference during movements taking place on the plane perpendicular to the camera axis. A frame of reference identifying X and Y axes as horizontal and vertical directions was manually set by an operator. In order to calibrate the space of interest, a plank of 324 cm with a perpendicular bar at the distal end (length 78 cm) was placed on three fixed points along the perimeter of the pool. A known length in the camera’s field of view and in the same plane as the movement was used as a measurement reference unit.

### Procedure

The trainer stood at the border of the pool facing the dolphins. Then by means of a fixed signal she asked one of the dolphins (i.e., “A”) to reach the stimulus and touch it with the rostrum (*Control* condition; Figure 2). As soon as the stimulus was touched the trainer called the dolphin back to the starting position by using a whistle. Then the trainer asked another dolphin (i.e., model) to reach towards, touch the stimulus and then return to the starting position. Immediately after, the trainer asked the dolphin “A” to perform again the reach-to-touch action (*Visuomotor Priming* condition). The “model dolphin” was kept oriented toward the platform by the trainer during performance of dolphin “A” to prevent it from observing its movements. Nonetheless, to exclude the possibility that in some trial “model” dolphin might be influenced by dolphin “A”, all its data were removed from the analysis. The alternating sequence was repeated 10 times per daily session, with the dolphin “A” always following the model (trials: 3, 5, 7, 9, 11) except for the control condition (trial 1). Both dolphins acted as models and experimental subjects, in randomized sessions for 6 days. Notably, only one of them acted as experimental subject during each daily session, since only one of them could perform the control condition (pre-observation of the model dolphin).

### Data Processing

Following data collection all videos were reviewed and segmented in order to extract the relevant reach-to-touch movements. For the “visuomotor priming” condition only movements in which the dolphin “A” observed the model performing the considered action before executing it were considered for subsequent analysis. Two independent reviewers who were unaware of the study rationale and blind to the experimental conditions scored each segment. They analyzed the footage frame-by-frame (frame duration: 20 ms) using an in-house software developed to perform* post hoc* kinematical analysis. Reliability between the two was quite high (Cohen’s *κ* = 0.86). A marker was placed *post hoc* on each subject’s rostrum. The starting position was defined as the water area frontal to the trainer’s platform at the border of the pool (2 m^2^). Movement onset was defined as the time at which the tangential velocity of the marker crossed a threshold (5 mm/s) and remained above it for longer than 500 ms. End of the movement was defined as the time at which the rostrum touched the stimulus. Movement tracking procedures were then performed in order to extract the kinematic parameter of interest. The analysis focused on the velocity profile to evaluate how the observer’s action changed following the observation of a model’s movements (Bisio et al., 2010). Average and Peak velocity, defined as the maximum velocity value occurring between movement onset and offset, were then extracted. Modifications of mean velocity in correspondence to changes of demonstrator’s velocity are usually considered proof of the occurrence of motion contagion.

### Statistics

Two mixed-design repeated measures ANOVAs were carried out on average and peak velocity with condition (control, visuomotor priming) as within-subject factor and dolphin identity (S., L.) as between-subjects factor, to control for possible inter-individual differences. The strength and direction of the linear relationship between average and peak velocity exhibited by dolphins “A” and their models for the visuomotor priming condition was determined by means of a correlation coefficient (Pearson’s *r*). In particular, we correlated the average and peak velocity of each trial of dolphin “A” to the just-observed movement of the model.

## Results

**Figure 3:**
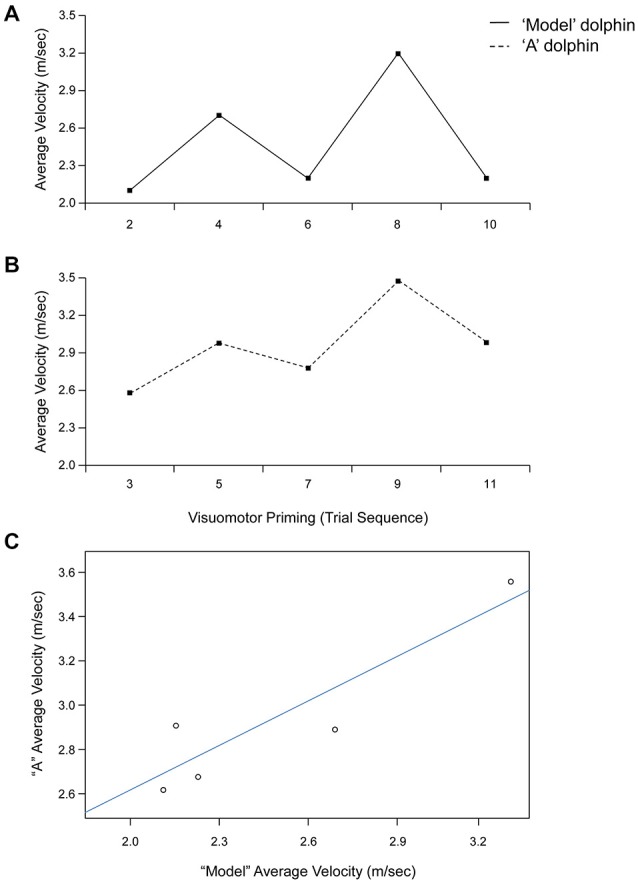
**Trial by trial average velocity (m/s) recorded from the model (upper panel, A) and “A” (lower panel, B) dolphins during a representative session (Day 1). (C)** Linear relationship between average velocity exhibited by dolphins “A” and model for the visuomotor priming condition at Day 1.

Statistical analyses revealed a significant effect of condition for both average (*F*_(1,4)_ = 8.67, *p* < 0.05, ${\text{η}}_{p}^{2}$ = 0.68) and peak (*F*_(1,4)_ = 7.94, *p* < 0.05, ${\text{η}}_{p}^{2}$ = 0.67) velocity. Dolphins were consistently faster after the visuomotor priming than for the control condition (Table 1). Notably, the interaction condition × dolphin identity was not significant (*p* > 0.05), suggesting that the priming influenced the motor performance for both dolphins. Motor contagion effects were confirmed by the high level of correlation between “A” dolphins and their models in terms of both average and peak velocity (Table 1; see Figure 3 for a representative session).

**Table 1:** **Average and peak velocity (m/s) mean values recorded from the “A” and the “model” dolphins**.

	Average velocity (m/s)				Peak velocity (m/s)			
Session	“A”		Model	*r*	“A”		Model	*r*
	Control	Visuomotor priming			Control	Visuomotor priming		
1	2.625	2.818	2.477	0.867*	8.062	8.781	8.409	0.941*
2	3.024	3.095	3.258	0.945*	6.345	7.071	9.740	0.879*
3	2.006	2.105	2.919	0.964**	7.387	8.062	9.662	0.983**
4	3.064	3.156	3.427	*0.823*	8.592	9.220	9.120	*0.856*
5	2.029	2.117	3.372	0.970**	8.902	9.220	12.042	0.858*
6	2.283	2.415	2.689	*0.837*	5.924	6.325	9.487	*0.845*

Pearson’s r indicate the correlation between each trial of dolphin “A” and the just-observed model movement. Asterisks indicate statistical significance (*p < 0.05; **p < 0.01).

## Discussion

Collectively, these findings provide definite evidence that dolphins considered in this study showed visuomotor priming effects, which might be due directly to activation by movement observation of motor representations coding topographically similar responses. These findings, therefore, offer the first convincing evidence that a non-primate species, the bottlenose dolphin, is capable of unintentional imitation.

Our study reveals interesting similarities between the way dolphins and humans respond to action observation. It suggests that action observation activates processes involved in action execution.

Synchronous behavior exhibited by many dolphin species are classical examples of on-line motor mimicry (Connor et al., 2000; Bauer and Harley, 2001). Rather our results, based on a *postponed* facilitation effect, suggest a representational system that encodes information about other’s behaviors in the same way it encodes information about one’s own behavior (Gopnik, 1993). These data fit well with theories assuming that the long-term S-R associations mediating unintentional imitation are products of learning (Heyes et al., 2005; Prinz, 2005; Catmur et al., 2009; Massen and Prinz, 2009). In this view, we cautiously suggest that motor representations activated following action observation might reflect an automatic resonance mechanism of motor structures paralleling observed movements. This would imply that the emergence of mirror-like manifestations are not a by-product of factors specific to primates, but instead might be attributable to general processes of associative learning fostered by a high degree of encephalization and cognitive ability. At the same time, the present results cannot exclude a speculative interpretation of unintentional imitation in non-primate species. Mirror neurons fire when monkeys perform an action, but also when the animal observe somebody else performing the same action (Gallese et al., 1996; Rizzolatti et al., 2001). Following this discovery, studies have provided some evidence that similar bimodal cells also exist in the human brain (Mukamel et al., 2010). Here, findings from our study might indicate the presence of a similar mirror mechanism in dolphins too.

In conclusion, we suggest that even when dolphins do not intend to imitate, the perception of action might activate the same neural (mirror neurons or mirror areas) or representational (common codes, shared representations or vertical associations) structures that are involved in the production of the perceived action. This might entail that in dolphins, rather than being distantly related by rules, the perception and the execution of action depend on the same systems, and the potential (or impulse) to produce an imitative action is generated not just when they intend to imitate, but whenever they watch another dolphin’s behavior.

To date, hypotheses about the evolution of unintentional imitation effects, as defined here, have largely confined on primates. Our findings show a striking case of convergence in the face of profound differences in neuroanatomical characteristics and evolutionary history. We are aware that the issue of whether the unintentional imitation effects exhibited by dolphins rely on similar mechanisms as primates remains partially unanswered, but this approach is promising and will shed new light in the debate on the “intentional” nature of imitative ability.

## Conflict of Interest Statement

The authors declare that the research was conducted in the absence of any commercial or financial relationships that could be construed as a potential conflict of interest.
